# A Simple Method for Assessing the Mental Health Status of Students in Higher Education

**DOI:** 10.3390/ijerph16234733

**Published:** 2019-11-27

**Authors:** Éva Bíró, Róza Ádány, Karolina Kósa

**Affiliations:** 1 Division of Health Promotion, Department of Preventive Medicine, Faculty of Public Health, University of Debrecen, H-4028 Debrecen, Hungary; kosa.karolina@sph.unideb.hu; 2 Department of Preventive Medicine, Faculty of Public Health, University of Debrecen, H-4028 Debrecen, Hungary

**Keywords:** assessment tool, mental health, likelihood ratio, predictive ability, students

## Abstract

Mental health problems are common among students in higher education all over the world, so identifying those who are at higher risk would allow the targeted provision of help. Our goal was to develop an assessment tool to identify students at risk for vulnerable mental health status. This tool was created from the 12-item General Health Questionnaire and Antonovsky’s abbreviated sense of coherence scale and was tested to distinguish between those with high or low mental resilience. Predictive ability was characterized by likelihood ratios taking the Beck Depression Inventory and perceived health as references. One-quarter (95% CI 21.1% to 29.7%) of the students had been in vulnerable mental health characterized by low sense of coherence and high distress, whereas 28.4% (95% CI 24.2% to 33.1%) seemed resilient, having high sense of coherence and low distress. The high negative predictive value of the assessment tool reliably identified resilient students in comparison with both the Beck Depression Inventory (98.6%) and perceived health status (83.9%). Use of the assessment tool is recommended for students to distinguish between those at decreased and increased risk in terms of mental health. Mental health services should be offered to students at higher risk.

## 1. Introduction

Early adulthood is a critical period in terms of developing the habits, self-image and social relations that are characteristic of individuals in adult life. Approximately three-fourths of all lifetime mental disorders start by the mid-20s, and the median age for substance disorders falls between 18 and 29 years of age [1].

Almost half of college-age youth had a psychiatric disorder according to an USA study, the most prevalent being some type of anxiety disorder. While the adjusted prevalence of substance use between college students and their non-college peers was no different, the former were significantly less likely to receive treatment for alcohol and drug use disorders compared to their peers not in college [2]. Another, more recent USA study found depression to be the most prevalent condition, with 17% of college students screening positive for depression [3]. Studies from countries other than the USA also support the notion that college students have a higher burden of mental distress [4] compared to their peers. This was shown among medical students in 29 studies conducted in Europe and English-speaking countries outside of North America [5]. A survey of 32% of colleges in the UK found an increase of students with disclosed mental health issues in the past 3 years [6]. Mental health problems have been prevalent in college students, including substance use, anxiety and mood disorders [7].

In spite of the increasing numbers of students suffering from mental health problems [8,9], attitudes towards seeking mental health services have become negative among American university students over the past 40 years [10]. Nearly one-fifth of college students thought that it was better not to disclose mental illness according to a recent study [11]. Moreover, current mental health practices are misguided [12] because they tend to focus only on those who already have mental disorders whereas the majority of new cases arise from the general population, so more effective mass population strategies should be followed [13]. That is, non-stigmatizing services should be offered to those not yet having manifest signs of a mental disorder who are most likely to benefit from them.

Early identification and stratification of youth at various levels of risk enables the development of targeted interventions. Resilient groups (those at decreased risk) could be identified by the salutogenic approach of Antonovsky which focuses on the generalized resistance resources of individuals facilitating their capacity to effectively cope with stressors. Antonovsky’s sense of coherence (SoC) reflects a person’s view of life and capacity to respond to stressful situations, contributing to health [14,15]. Strong positive relation between SoC and perceived good health as well as mental health [16,17] and physical health [18] have been uncovered. The salutogenic approach could be applied in universities by focusing attention to vulnerable students while also offering help to resilient and effectively coping students in case they need it [19].

Mental health problems, if left unrecognized and untreated, may result in failed exams or dropping out, and even in attempted or completed suicide as well as engagement in risky behaviors leading to serious injury, disability, or death [20]. Identification of resilient students as opposed to those who exhibit signs of mental strain at an early stage would enable a more effective use of limited resources by addressing problems as early as possible among those in greatest need to prevent the development of full-blown mental problems.

Based on the results of previous [21,22] and present surveys, and in line with the recommendation of Huppert regarding the use of an universal approach to reduce the number of people with mental disorders [12], we aimed at developing an assessment tool to identify vulnerable as well as resilient college students in terms of mental health problems.

## 2. Materials and Methods

### 2.1. Study Population

A cross-sectional study was carried out among students of public health, nursery education, and physiotherapy of the University of Debrecen, Hungary in 2008, 2009 and 2010, respectively. The study population consisted of all full-time students registered at the courses of public health (*N* = 194), nursery education (*N* = 168) and physiotherapy at years 1–3 (*N* = 153).

Data of the general population were taken from a mental health survey representative of the adult Hungarian population that was carried out in a sample of 1200 persons by the Median Polling Institute in 2010 following a multistage stratified cluster sampling.

### 2.2. Data Collection

The timing of data collection on mental health is critical among university students because their stress level fluctuates during the academic year. A potential source of bias might be excessive stress close to or during the exam period, so data were collected in mid-semester in order to reduce this type of bias. Each student was invited in person after class to fill a paper-based, self-administered, anonymous questionnaire. The distribution and recollection of questionnaires was carried out by student volunteers from courses not involved in the survey in order to avoid any pressure for participation.

The research was carried out in accordance with the Helsinki Declaration. Ethical permission was issued by the Regional and Institutional Commission on Research Ethics of the Medical and Health Science Centre of the University of Debrecen, Hungary (DEOEC RKEB/IKEB: 2506-2006). The students were informed in writing and in person that participation was anonymous and voluntary, and they had the right to refuse to participate. The participants had given verbal consent for their data to be used in the research. No personal data were collected so a consent form was not requested to be signed by the ethics committee.

#### Scales of the Questionnaire

The questionnaire was similar to those used in previous surveys among medical students [22] and future teachers [21] and included scales on mental health (sense of coherence, psychological distress, perceived stress, depression, social support; the scales for perceived stress and depression were not included in the questionnaire for public health students), perceived health, health locus of control (how much can do for own health), demographic (age, sex, residence) and socioeconomic data (parents’ educational level, family’s economic status). Items not referred separately were taken from the tool of the Hungarian National Health Interview Survey (HNHIS) of 2003 [23]. The full questionnaire took around 15–20 min to fill.

Sense of coherence was measured by the abbreviated 13-item scale (SoC-13) [24] which had been validated in Hungarian [25]. Items were answered on a 7-point Likert scale; the total score varied between 13 and 91. Higher scores indicate greater sense of coherence.

The 12-item version of the General Health Questionnaire (GHQ-12) was used to detect psychological distress. Questions were answered on a 4-point Likert scale. Cases were detected by scoring in the simplest manner [26] which assigns a score of 1 to each symptom, while lack of a particular symptom is scored by 0 so that the total score varies between 0 and 12. Total score above 4 was set as the threshold indicating notable psychological distress, identical to that used in the Hungarian National Health Interview Survey of 2003 [27].

The 9-item Hungarian version of the Beck Depression Inventory (BDI) was used to assess depression [28]. Scores below 9 indicated no depression; scores between 10 to 18 indicated mild depression; scores between 19–25 indicated moderate depression; scores above 25 estimated severe depression.

The questionnaire of the mental health survey of the general population included demographic items as well as the SoC-13 and the GHQ-12 scales.

### 2.3. Statistical Methods

Data were entered into a Microsoft Excel database. Data entry as well as the full database were checked and cleaned by removing inconsistent answers. Data from all three student groups (public health, nursery education, physiotherapy) were merged into one database and analyzed together. Intercooled Stata 10.0 for Windows was used for data transformation and analysis. Categorical variables were analyzed by the chi-squared test and Fisher’s exact test. Results were compared to that of a representative survey of the Hungarian adult population using the two-sample test of proportion.

Available case analysis was used since less than 5% of the data were missing for all variables, and data were assumed to be missing completely at random based upon the result of Little’s test (*p* = 0.401). Sensitivity analysis was not performed for handling of missing data.

## 3. Results

### 3.1. Demographic and Socioeconomic Data

A total of 412 of the potentially eligible 515 students were present at the time of the data collection and all of them agreed to participate in the study. Three records were deleted after data checking because less than one-quarter of the questions were filled out. The overall response rate was 79.4% (*n* = 409). Mean age of the sample was 20.81 years (SD: 1.99; min. 18, max. 41); the majority (98.5%) being under 26 years of age. All three courses were dominated by females, but their proportion was significantly lower among public health students (83.5%) compared to the two other groups (students of nursery education, 96.9%; physiotherapy, 93.1%; *p* < 0.05). The sex ratio was representative of the students at these courses. Respondents were representative by study year for each course. Detailed demographic and socioeconomic data are shown in Table 1.

### 3.2. Creating a Composite Indicator for Assessment

The basic concept of the assessment tool was that it should assess both the positive and the negative aspects of mental health in a non-stigmatizing manner. GHQ has been widely used as a screening device for identifying minor psychiatric disorders in the general population, but it reflects a deficit-based approach to mental health. Antonovsky’s salutogenic concept is oriented to resilience, so combining the two tools provides a more balanced assessment of mental status.

A composite indicator of mental well-being was created from sense of coherence (*n* = 399) and psychological distress (*n* = 402) to assess mental health. For SoC, there is no threshold below which it could be considered too low or not normal, therefore—in line with previous studies [29,30,31,32,33]—after defining tertiles of the total score, SoC scores at the highest tertile (above 65 points) were considered normal, whereas scores in the two other tertiles as low. As to psychological distress, the cut-off value of 5 points was used to identify those who were not distressed. A composite indicator for mental status was created from the categories of SoC and GHQ using a 2 × 2 table with the following categories: (1) resilient (normal SoC, normal GHQ); (2) vulnerable (low SoC, high GHQ); (3) non-classifiable: normal SoC and high GHQ; or (4) non-classifiable: low SoC with normal GHQ (Table 2).

In order to check the predictive ability of our assessment tool, positive (LR+) and negative (LR−) likelihood ratios were calculated based on the Bayes theorem considering the BDI score and perceived health as reference. The BDI-9, SoC-13 and GHQ-12 scores were available for 256 students; perceived health, SoC-13 and GHQ-12 were known for 393 students. BDI categories reflecting moderate and severe depression (over 18 points) were combined to identify those at-risk (mentally vulnerable); those who scored below 19 points were defined not at risk after Kopp and her co-workers [28]. Perceived health was dichotomized into categories of having good or very good vs. fair or less than fair perceived health. LR was used because it does not require dichotomization so all four categories (including the two non-classifiable risk categories with conflicting SoC and GHQ results, Table 2) were used to calculate the LR. Nonparametric ROC analysis was performed to calculate the area under the curve (AUC).

### 3.3. Mental Status of the Students Assessed with a New Composite Indicator

The assessment capacity of the composite indicator created from sense of coherence and psychological distress was tested as described above. Based on the assessment tool, four groups of students could be identified, as shown in Table 3, of which two groups showed congruent results according to GHQ-12 and SoC-13 alike. More than one-quarter (28.4%, 95% CI 24.2% to 33.1%) of students could be defined as being resilient, that is, having good mental health with normal sense of coherence and low distress. Vulnerable students had low sense of coherence along with high levels of distress, comprising one-quarter (25.1%, 95% CI 21.1% to 29.7%) of the participants. Two other groups with inconsistent results captured 46.5% of the students: 44.2% of them had low sense of coherence and normal levels of distress; 2.3% had normal sense of coherence and high levels of distress. There was a significant gender difference in resilience (normal SoC, normal GHQ) with almost twice as many resilient males (43.2%, 95% CI 28.2% to 59.6%) compared to females (27.0%, 95% CI 22.7% to 31.9%; *p* = 0.038).

### 3.4. Mental Status of the Students Compared to the General Population

The proportion of vulnerable students (low SoC and high GHQ) was compared to 18- to 25-year-olds in the general population and was found to be three times higher (25.1% vs. 7.9%; *p* < 0.001) (Table 4). There was no significant gender difference regarding the proportion of those at risk in the general population (males: 9.8%, females 6.4%; *p* = 0.559) and there was also no difference between at-risk male students and their peers in the general population (13.5% vs. 9.8%; *p* = 0.605). The proportion of vulnerable female students was nearly four times higher compared to their peers in the general population (26.2% vs. 6.4%; *p* = 0.003).

### 3.5. Predictive Ability of the Composite Indicator

The LR+ of the composite indicator was 2.61, meaning that the prevalence of vulnerable mental status was 2.61 times higher among depressed students compared to students who were not depressed (positive post-test probability = 0.405). LR− was 0.054 producing a negative post-test probability of 0.014. Accordingly, students who scored depressed by the BDI would be 18 times less frequently categorized as resilient by the composite index compared to those without symptoms of depression. The positive predictive value of the assessment tool calculated with the above data proved to be 40.5% as opposed to the negative predictive value of 98.6%. The LR for those who had low SoC and normal stress was 1.05 showing that this combination is more frequent among depressed students. The AUC was 0.743 (95% CI 0.683–0.803).

The predictive ability of the new assessment tool against BDI was compared with the predictive ability of the GHQ and SoC alone. For GHQ-12, the positive predictive value was 36.6% while the negative predictive value was 86.8%. For SoC-13, the positive predictive value was 29.6% and the negative predictive value was 98.8%.

Among students with bad subjective health, the prevalence of vulnerable mental status was 2.6 times higher compared to students who were in good health (LR+ = 2.60, positive post-test probability was 0.606). LR− was 0.32 producing a negative post-test probability of 0.161. This shows that students in bad subjective health would be 3 times less frequently categorized as resilient by the composite index compared to those with good health. The positive predictive value of the assessment tool calculated with the above data proved to be 60.6% as opposed to the negative predictive value of 83.9%. The LR for those who had low SoC and normal stress was 1.02 showing that this combination is more frequent among students with bad health. The AUC was 0.687 (95% CI 0.637–0.736).

The predictive ability of the new assessment tool against bad subjective health was compared to the predictive ability of the GHQ and SoC alone. In case of GHQ, the positive predictive value was 58.3%, while the negative predictive value was 70.9%. For SoC, the positive predictive value was 45.9% and the negative predictive value was 82.6%.

## 4. Discussion

Using the new assessment tool, nearly 30% of students were identified as resilient with normal SoC and low GHQ. Mental health was considered worrisome for those who had low SoC and high GHQ. This vulnerable category captured almost one-quarter of the study population, which is in line with another study where the proportion of those who had any mental health problem was similar (33.8% [34]).

Students with normal sense of coherence and low levels of mental distress can be considered mentally resilient according to our assessment tool. Of all resilient students, 98.6% were identified as not depressed by the Beck Depression Inventory. Of those who were defined mentally vulnerable (with low sense of coherence and high levels of mental distress), only 40.5% scored as depressed by the BDI. There was not one student in the high BDI score group who would have had high SoC and high stress, pointing to the protective effect of higher levels of SoC in relation to depression even in distressed people. Of the resilient students, 83.9% perceived their health as good.

Our assessment tool can reliably distinguish between students who are in reasonably good mental health or can be considered resilient, and those who are at increased risk and need further attention and targeted support during their studies. Our assessment tool can be reliably used to identify vulnerable students in a reasonably simple and non-stigmatizing manner that allows the provision of timely and targeted support and help during their studies.

### 4.1. Strengths and Limitations

An advantage of the present survey was its wide scope (inclusion of all students of three courses) and high response rate that was representative of the students by sex and study year.

The timing of data collection on mental health is critical among university students because their stress level strongly fluctuates during the academic year. In order to avoid measuring further increased stress before or during the exam period, data were collected in mid-semester when stress related to the examination period is at its lowest.

A considerable strength of our assessment tool is that it approaches mental status not only from a negative (deficit) but also from a positive (resource) perspective. The salience of our approach is reflected by identifying only one depressed student among those who had a normal sense of coherence (1.3%).

Our assessment tool has a low negative post-test probability, that is, high negative predictive value. However, predictive ability is limited by the fact that calculations were based on a reference test (BDI) with screening rather than diagnostic features. Nevertheless, BDI demonstrates a high internal consistency, with alpha coefficients of 0.86 and 0.81 for psychiatric and non-psychiatric populations, respectively [35]. The predictive ability of this assessment tool is more favorable compared to using the GHQ or SoC alone.

### 4.2. Mental Health Screening in Practice

The new assessment tool measuring sense of coherence and psychological distress could be offered to students in an anonymous manner, followed by evaluation and individualized online messages on recommended services and support options. Compared to the validity of other similar screening tests, the features of our assessment test are acceptable, especially if compared to the depression screening tool (BDI). The screening for depression in adults over 18 years of age is recommended by the US Preventive Services Task Force [36]. However, the positive predictive value of BDI was 54% and the negative predictive value was 99% in a study in which the prevalence of major depression was similar to that in our study [37]. Furthermore, comparing our results to a previous study where GHQ-12 alone was used to detect depression, both the positive (40.5% vs. 27.8%) and the negative (98.6% vs. 97.1%) predictive values of our assessment tool were higher [38].

Time alone does not seem to solve mental problems in college students as they tend to persist even after 2 years of follow-up [39]. There have been initiatives for reaching out to college students with mental problems, but these focused on depression [40,41] and the prevention of suicide [42].

In another type of initiative aiming at increasing the uptake of clinical services, the University of Washington developed a web-based system for students to self-screen for anxiety, depression, attention-deficit/hyperactivity disorder, alcohol use and eating disorder. The system logged more than 2700 visits, 1003 screening sessions and 438 referral requests in 17 months showing that such a system can increase care-seeking. However, the system requires an elaborate IT background with secure data repositories and processes of data exchange that must be supported by a sufficiently staffed primary care center capable of handling all incoming referrals [43].

Currently there is no recommendation for the screening of vulnerable students in higher education. Screening only for depression among students would miss many in need of help, such as those struggling with anxiety or substance abuse [7].

## 5. Conclusions

Since mental health problems are common among those in helping professions, and mental health problems burden students disproportionately more than their peers [22,44,45], the best time to take action seems to be during their years in college. Considering the help-avoiding attitude of students [46] and the perennial lack of resources to deal with mental problems at almost all institutes of higher education, we propose the assessment of mental health of students using the GHQ-12 and SoC-13 scales combined as described above. The tool has the advantage of having a balanced focus on mental health from an aspect of vulnerability as well as of resilience; it reliably separates those who are psychologically definitely at risk from those who can be considered reasonably resilient. Moreover, it is simple to use and avoids stigmatization. The test could be easily adapted to an online format, and based on its results, respondents could get detailed personalized advice on available supportive services, tailoring the message to their level of risk. A computerized risk assessment followed by personalized message would yield immediate help and might facilitate the uptake of preventive services while avoiding stigmatization and the overburdening of preventive services. An online mental health support system can be a viable alternative or supplement to university counselling services [47,48]. Routine administration of our assessment test would enable the monitoring of mental problems among college students and enhance seeking help, diagnosis and treatment as recommended [7].

## Figures and Tables

**Table 1:** Demographic and socioeconomic data of the participants.

Students by Course	No. of Students			Mean Age (Years; Standard Deviation; Min–Max)	Proportion of Females (%)	Grade and Level
	Potentially Eligible	Included in the Study	Analyzed			
Public health students	194	149	146	20.61 (1.53; 18–25)	83.5	1–3 years (Bachelor), 4–5 years (5-year program)
Nursery education students	168	133	133	19.98 (1.16; 18–24)	96.9	1–3 years (Bachelor)
Physiotherapy students	153	130	130	21.86 (2.56; 19–41)	93.1	1–3 years (Bachelor)
**All students**	**515**	**412**	**409**	**20.81** **(1.99; 18–41)**	**90.9**	

Features of the total sample are marked with bold.

**Table 2:** Creation of a composite indicator for assessing mental status in students.

Measures of Mental Health		Sense of Coherence	
	Categories	Low: at the 3rd (Lowest) and 2nd Tertiles	Normal: at the 1st (Highest) Tertile
**Psychological Distress**	high: at or above the cut-off for distress	mentally vulnerable	non-classifiable
	normal: below the cut-off	non-classifiable	mentally resilient

**Table 3:** Mental well-being of students assessed with the composite indicator.

Measures of Mental Health		Sense of Coherence (%)	
	Categories	Low	Normal
**Psychological Distress (%)**	high	25.1 (vulnerable)	2.3
	normal	44.2	28.4 (resilient)

**Table 4:** Mental well-being of 18–25-year-old Hungarian adults assessed with the composite indicator.

Measures of Mental Health		Sense of Coherence (%)	
	Categories	Low	Normal
**Psychological Distress (%)**	high	7.9 (vulnerable)	0
	normal	60.2	31.8 (resilient)
